# Effectiveness of personal genomic testing for disease-prevention behavior when combined with careful consultation with a physician: a preliminary study

**DOI:** 10.1186/s13104-018-3330-9

**Published:** 2018-04-03

**Authors:** Mikihiro Hayashi, Atai Watanabe, Masaaki Muramatsu, Naohide Yamashita

**Affiliations:** 1 Department of General Medicine, School of Medicine, Juntendo University, 2-1-1, Hongo, Bunkyo-ku, Tokyo, 113-8421 Japan; 2 The Cancer Institute Hospital of JFCR, Tokyo, Japan; 3 Department of Molecular Epidemiology, Medical Research Institute, Tokyo Medical and Dental University, Tokyo, Japan; 4 Institute of Medical Science, University of Tokyo, Tokyo, Japan

**Keywords:** Personal genomic testing (PGT), Direct-to-consumer (DTC), Disease prevention, Genetic counseling

## Abstract

**Objectives:**

There are many direct-to-consumer (DTC)-type personal genomic testing (PGT) services commercially available to the public, providing the specific disease susceptibilities of individuals. While these services do not appear to stimulate disease-prevention behavior, few studies have addressed the methods to do so. We investigated the effectiveness of combining a consultation with a physician with the delivery of test results from a DTC-type PGT, as a preliminary study to identify the effective genomic testing for disease-prevention. A prepared physician disclosed the PGT results of twenty healthy subjects and provided a specific consultation on the high-risk diseases for each subject. The effects on the sense of health, understanding of possible future diseases, and preventive behaviors for each subject were examined pre-PGT, post-PGT, and 3, 6, and 12 months post-PGT.

**Results:**

Significant increases between the pre- and post-PGT scores were observed for the awareness of lifestyle effects on developing those diseases (P < 0.05) and the awareness of the ability to influence disease onset (P < 0.01). The follow-up questionnaire results showed that over 60% of the subjects changed their lifestyles in favor of disease prevention. These results suggest that combining the DTC-PGT with a careful physician consultation may be effective at motivating people toward preventive behavior.

**Electronic supplementary material:**

The online version of this article (10.1186/s13104-018-3330-9) contains supplementary material, which is available to authorized users.

## Introduction

Many susceptibility tests for common diseases, based on the analysis of common single-nucleotide polymorphisms (SNPs) in individuals, are marketed directly to consumers. Personal genomic testing (PGT) using such tests has long been expected to contribute to the personal motivation to prevent disease. However, recent meta-analyses do not support the hypothesis that DNA-based risk estimates for disease occurrence motivate risk-reducing health behaviors [1, 2].

Another study of disease risk effects in a 2037-subject cohort showed that direct-to-consumer (DTC)-type PGT tests did not lead to any measurable short-term changes in diet, exercise, or use of health-screening tests [3]. Existing evidence is unconvincing that DNA-based risk assessments motivate people to take clinical measures that reduce disease risks, such as surgery, even when the subject is at high risk for a disease [4].

These observations may be due to the low clinical validity and utility of DTC-type genomic testing, which have long been criticized, but no consensus exists so far, regarding which aspects of a DTC service should jeopardize its permissibility [5–7], and consumers are undergoing DTC-type PGT in significant numbers [8–10].

While it has been pointed that the explanations attached to the test results produced by commercially available DTC tests usually appear to be insufficient [11, 12] and that DTC-type PGT requires tailored follow-up to be effective, few studies have focused on the means of delivering information on DTC-type test results [1].

We therefore conducted planned PGT, taking a detailed family history for each subject, accompanied by a prepared consultation with a physician, to examine how this structured testing process would affect the participants’’ thoughts on lifestyle and disease prevention. While this study was conducted as the preliminary stage of a follow-up study of PGT using a more detailed analysis of each personal genome, including exome sequencing, it aimed to investigate the effectiveness of providing a physician consultation with the delivery of PGT results on participant’s perceptions of lifestyle changes and disease-prevention activities.

## Main text

### Methods

#### Design

The study was a preliminary cross-sectional exploratory study that used questionnaires completed before and after the PGT. The longitudinal study at 3, 6, and 12 months after the PGT was also conducted using another questionnaire.

#### Approach and recruitment

**Fig. 1:**
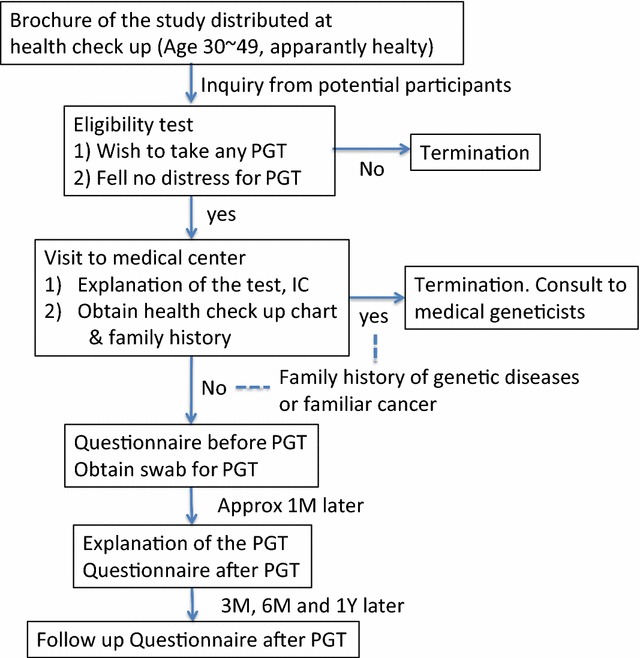
Study profile

The overall workflow of the study is depicted in Fig. 1.

From Sep 2014 to Mar 2015, by distributing the introductory brochures at three companies and the University of Tokyo Institute of Medical Science, we recruited the subjects who were interested in PGT and who were assessed as being healthy during a checkup. The eligibility criteria were (1) apparently healthy men or women aged 30–49 years, with (2) an interest in undergoing PGT. An online questionnaire was provided to all who inquired (Additional file 1) to select the participants who wanted to learn about genetic risks and did not feel distressed by the possible results, regardless of the curability of the disease.

#### Procedure

Each participant was invited to visit our office to receive a detailed explanation of the entire study procedure by a prepared general physician (N.Y.) and asked to provide written informed consent. Prior to enrollment, each participant was also required to provide a recent annual medical health checkup chart to confirm that there were no significant signs of diseases. Next, the detailed family history of the participant was taken. Those who had any family history of genetic diseases or cancer were excluded from the study and introduced to the relevant heredity clinic at the University of Tokyo Institute of Medical Science.

Next, each participant was asked to complete a pre-PGT questionnaire, which was composed of 30 multiple-choice questions, designed to measure the perceived levels of health and future disease risk (Additional file 2). These questions were selected and modified from SF-36 [13, 14] and the Brief Illness Perception Questionnaire (B-IPQ) [15].

Then, a saliva sample was collected for DNA analysis and the PGT (Additional file 3) was conducted to report the risks for 60 diseases and constitutional phenotypes, calculated by using 1–12 reported single nucleotide polymorphisms (SNPs) previously published in the Japanese or Asian population, using a method commonly utilized for this type of test [16].

The PGT report was ready in 1–3 months, at which time each participant was asked to revisit the medical center.

The same general physician (N.Y.) disclosed the report to each participant face-to-face, and provided the consultation, which included explanations regarding those diseases for which the participant’s relative risk was estimated to be greater than 1.5, as well as the explanations of disease-prevention methods currently considered to be scientifically sound. The explanation session took 1 h on average. Following the consultation with the physician, participants were asked to fill out a post-PGT questionnaire (identical to the pre-PGT questionnaire, Additional file 2).

Three, six, and 12 months after the PGT, another questionnaire, which was designed to record the individuals’ sense of health, understanding of possible future diseases, and disease-preventive behaviors (Additional file 4), was delivered to participants by e-mail, and responses were returned by e-mail. These questions for the follow-up questionnaire were designed based on previous studies [1, 3].

#### Statistical analysis

The pre- and post-PGT questionnaire scores were analyzed by paired t test using the Macplus statistical software (Apple Inc., Cupertino, CA). The follow-up questionnaire was analyzed on a narrative basis.

### Results

#### Participation

In response to the distributed brochure, 34 subjects expressed interest in participating in the study via e-mail. Each subject was asked questions via a web survey tool, and those who felt any distress regarding the study procedures were excluded. Twenty subjects (9 men, 11 women, mean age 38.7 years) were enrolled in the subsequent study phase.

Upon the initial visit of a subject to the medical center, no exclusion was made based on the health checkup or the family history interview. After informed consent was received, a saliva sample was collected from the subject, and the DNA was analyzed. All 20 subjects filled out the pre- and post-PGT questionnaire, as well as the follow-up questionnaires.

#### Questionnaire results

In the pre- and post-PGT questionnaire, the Q1 section, composed of four questions, scored each respondent’s perception of their current health status. These scores were not significantly different between the pre- and post-PGT responses.

The Q2 section of the questionnaire pertained to how the subject thought their health would be in 10 years. The numbers of subjects who felt that they would be generally healthy and would manage their daily life did not significantly differ between the pre- and post-PGT responses, which were 17 and 15, respectively, out of 20 subjects.

The Q3 section of the questionnaire, composed of six questions, asked how the respondent would feel were they to become ill in 10 years. As shown in Table 1, there were significant differences in the scores for three questions regarding how well subjects thought they would be able to control illness (P < 0.01), how much their lifestyle and attitude would be able to affect illness (P < 0.05), and how well they would be able to understand the diseases (P < 0.01). There were no differences in how long they thought they would be ill or in how much emotional distress they anticipated they would feel.

**Table 1:** Answer summary for Q3 section of the questionnaire; Pre- and Post-PGT questions, designed to measure the perceived levels of health and future disease risk

Q3	Mean score before PGT^a^	Mean score after PGT^a^	Paired T test
1. If you become sick in 10 years’ time, how long do you think it would last?	4.75	4.65	NS
2. If you become sick in 10 years’ time, how much do you think it affects you and your family?	5.8	5.1	NS
3. If you become sick in 10 years’ time, how well do you think you could control your illness?	5.75	7.1	P < 0.01
4. How well does your lifestyle and life attitude help prevention of the diseases you might get in 10 years’ time?	4.75	6.4	P < 0.05
5. How well do you think you understand the diseases you might suffer from in the future?	5.65	8.1	P < 0.01
6. How much emotional distress (such as anger, fear, anxiety, depression) would you feel when you think that you may be ill in 10 years’ time?	3.55	3.75	NS

*NS* not significant

^a^ The scores are distributed from 0 to 10

For the question 3–4, “How well does your lifestyle and attitude help to prevent the diseases you might acquire in 10 years?,” the pre-PGT response mean was 4.75 (SD 2.7), post-PGT response mean was 6.4 (SD 2.59), and the effect size, d, was 0.62, with a sample size of 20. At α = 0.05, and 1 − β = 0.8, the post hoc power was calculated to be 0.84. If we assume the effect size, d, of the following study to be 0.5, with the a priori power to be 0.8, the sample size, n, would be calculated to be 27. If we conservatively assume the effect size, d, to be half of the result of this study, 0.31, the sample size, n, would be 66. These calculations were performed by G*Power Ver3.1.

The Q4 section of the questionnaire, composed of 18 questions, pertained to what the subject felt would affect their future health or illness (in 10 years’ time). The post-PGT scores were significantly higher than the pre-PGT scores in the areas of inappropriate medication (P < 0.05), environmental pollution (P < 0.01), negative thinking (P < 0.05), drinking (P < 0.05), and smoking (P < 0.05) (Additional file 5).

#### Follow-up questionnaire

As shown in Table 2, in follow-up questionnaires completed 3, 6, or 12 months after the PGT, 70, 60, or 80% of the participants, respectively, reported lifestyle improvements.

**Table 2:** Answer summary of the follow-up questionnaire

Q#		3 month later	6 month later	12 month later
1	Recall the results	20	19	20
2	Been consulted by experts	16	14	14
3	Changed the life-style behavior	14^a^	12^a^	16^a^
4	Changed diet	12	14	17
5	Changed exercise	12	10	15
6	Reduced alcohol and/or smoking	10	8	11
7	Started any healthy habit	6	3	12
8	Started anything for health (specifically)	6	2	5
	Changed any behavior (any YES for Q-4 to 8)	17^b^	15^b^	19^b^

Number of the participants who answered YES for each question. All 20 participants completed the questionnaire each time

^a^ There were some subjects who answered no to Q-3 and answered yes to Q-4 to 8

^b^ This number reflects those who answered YES to any question from Q3 to Q8

Among the 20 subjects, 14 answered that they had changed their lifestyle behavior at 3 months, 12 at 6 months, and 16 at 12 months after the PGT. In 12 months, 17 of the participants had changed their diet, 15 had changed their exercise habits, and 11 had changed their alcohol ingestion and/or smoking. After 12 months following the PGT, 19 subjects out of 20 reported some type of behavioral change.

The obtained free responses to question 9 included “I have become more diligent at taking the time to chew my food well”; “I have adopted the habit of not snacking between meals but rather eating more vegetables”; and “I became more conscious of my health conditions”. None of the subjects exhibited mental problems. The only negative psychology-related comment was from one subject who stated, “I feel a little controlled by the PGT result, but that is not a serious issue for me”.

### Discussion

Our initial hypothesis was that receiving PGT results accompanied by a consultation from a medical doctor would positively affect an individual’s perceptions of health and illness. We observed no significant effects in how the subjects perceived their current health. The PGT also did not affect their feelings on whether they would become ill in the future. However, as shown by Q3-5 (Table 1), the subjects responded that they gained an understanding of the diseases that they might suffer from in the future. Interestingly, there was a significant difference in how much control they felt that they would have over future illnesses (Q3-3). They also believed that their lifestyle and attitude might help to prevent these potential diseases (Q3-4). These results can be interpreted to suggest that receiving PGT results with appropriate counseling could boost a consumer’s confidence in their responsiveness to future diseases. The responses for Q4 (Additional file 5) indicated that participants tended to ascribe greater weight to environmental factors than to genetic factors for achieving good future health. This result indicates that the physician’s counseling helped participants understand the roles of genetic and environmental factors in developing common chronic diseases.

In follow-up investigations at 3, 6, and 12 months after PGT, more than half of the subjects reported improved lifestyles. After 12 months of PGT, we found that 95% of the subjects reported some type of behavioral change for disease prevention and no subjects reported serious mental health problems. A prepared PGT consultation with a physician discussing disease risks and prevention strategies enabled participants to adopt disease-prevention-related behaviors without experiencing any significant adverse effects.

The conjunction of PGT and professional counseling may empower people to adopt healthier lifestyles that help mitigate their personal disease risks.

### Conclusion

By combining the PGT with a physician consultation, we found that the subjects reported a significantly greater understanding of the diseases they were at risk for in the future, increased confidence in their ability to control the future of these diseases and the adopted improved lifestyles. Given the small and non-representative sample, the findings are not definitive, but justify a larger trial.

## Limitations

### Subjects

The subjects were twenty Asians, which did not meet the expected number of participants, according to the statistical power we calculated. This pool should be expanded to increase the reliability of our results, and there is some sampling bias as the subjects were limited to those who self-reported no distress at the prospect of learning of disease risks, regardless of curability.

### DNA testing and family history recording

In this study, the susceptibility to diseases was assessed for each subject based on common SNPs and family history. As many heritable characteristics are still ‘missing’ [17], some rare variants might be included in the following study, and a more systemized methods of obtaining family history would improve the predictivity.

### Communication style of the physician

In this study, a prepared general physician (N.Y.) explained the PGT results to each participant and provided a consultation in person. While the communication process has been noted to be important in DNA testing [18], it is difficult to standardize. A learning program and/or manualized material for other physicians should be considered.

## Additional files

**Additional file 1.** Screening Questionnaire.

**Additional file 2.** Pre- and post-PGT Questionnaire.

**Additional file 3.** Used PGT.

**Additional file 4.** Follow-up Questionnaire.

**Additional file 5.** Answer summary for Q3 section of the Pre- and post-PGT questionnaire.

## Abbreviations

DTC: direct-to-consumer
PGT: personal genomic testing
SNPs: single-nucleotide polymorphisms
B-IPQ: Brief Illness Perception Questionnaire
