# Supplementary material for: Effectiveness of personal genomic testing for disease-prevention behavior when combined with careful consultation with a physician: a preliminary study
Source: BMC Res Notes. 2018 Apr 3;11:223. doi: 10.1186/s13104-018-3330-9 (PMC5883259; doi:10.1186/s13104-018-3330-9)
Supplement: Supplementary file 1 — Additional file 1. Screening Questionnaire. [file 13104_2018_3330_MOESM1_ESM.docx]

**Additional File 1**

Screening Questionnaire;

1. Do you feel distress for receiving a genetic test of yourself?
2. Do you want to know your genetic risk for diseases even if there is no remedy to prevent your disease?
