# Supplementary material for: Effectiveness of personal genomic testing for disease-prevention behavior when combined with careful consultation with a physician: a preliminary study
Source: BMC Res Notes. 2018 Apr 3;11:223. doi: 10.1186/s13104-018-3330-9 (PMC5883259; doi:10.1186/s13104-018-3330-9)
Supplement: Supplementary file 2 — Additional file 2. Pre- and post-PGT Questionnaire. [file 13104_2018_3330_MOESM2_ESM.docx]

**Additional File 2**

Pre- and Post-PGT Questionnaire;

Q1. This questionnaire is to ask how you feel about your current health. Please indicate to what extent you agree or disagree with the following statements about answering research questionnaires.

1. I am susceptible to illness more than an average person.
2. I completely agree.
3. 1 agree
4. I am neutral
5. I don’t agree
6. I don’t agree at all
7. I am as healthy as other people.

1) I completely agree.

2) I agree

3) I am neutral

4) I don’t agree

5) I don’t agree at all

1. I feel like my health will decline.

1) I completely agree.

2) I agree

3) I am neutral

4) I don’t agree

5) I don’t agree at all

1. My health is in a very good condition.

1) I completely agree.

2) I agree

3) I am neutral

4) I don’t agree

5) I don’t agree at all

Q2. This question is to ask how you think about your health in ten years’ time. Select the most appropriate.

1) I will be totally healthy in ten years’ time.

2) I might be unhealthy but I could manage my daily life.

3) I may be sick and could not manage my daily life

4) I might be sick and be hospitalized.

Q3. Select the most appropriate answer (check parenthesis).

1. If you become sick in ten years’ time, how long do you think it would last?

Last Last Last Last Last

very short relatively short temporarily fairly long life time

0 1 2 3 4 5 6 7 8 9 10

| □ | □ | □ | □ | □ | □ | □ | □ | □ | □ | □ |
| --- | --- | --- | --- | --- | --- | --- | --- | --- | --- | --- |

1. If you become sick in ten years’ time, how much do you think it would affect you and your family?

No effect severely affected

0 1 2 3 4 5 6 7 8 9 10

| □ | □ | □ | □ | □ | □ | □ | □ | □ | □ | □ |
| --- | --- | --- | --- | --- | --- | --- | --- | --- | --- | --- |

1. If you become sick in ten years’ time, how well do you think you could control your illness?

I may be I can I can completely

Not at all somewhat able to control reduce susceptibility control

0 1 2 3 4 5 6 7 8 9 10

| □ | □ | □ | □ | □ | □ | □ | □ | □ | □ | □ |
| --- | --- | --- | --- | --- | --- | --- | --- | --- | --- | --- |

1. How well does your lifestyle and life attitude help prevention of the diseases you might get in ten years’ time?

Entirely useless about half very useful

0 1 2 3 4 5 6 7 8 9 10

| □ | □ | □ | □ | □ | □ | □ | □ | □ | □ | □ |
| --- | --- | --- | --- | --- | --- | --- | --- | --- | --- | --- |

1. How well do you think you understand the diseases you might suffer from in the future?

No need to I don’t understand I understand

understand much about it very well

0 1 2 3 4 5 6 7 8 9 10

| □ | □ | □ | □ | □ | □ | □ | □ | □ | □ | □ |
| --- | --- | --- | --- | --- | --- | --- | --- | --- | --- | --- |

1. How much emotional distress (such as anger, fear, anxiety, depression) would you feel when you think that you may be ill in ten years’ time?

No emotional Some emotional Much emotional

distress distress distress

0 1 2 3 4 5 6 7 8 9 10

| □ | □ | □ | □ | □ | □ | □ | □ | □ | □ | □ |
| --- | --- | --- | --- | --- | --- | --- | --- | --- | --- | --- |

Q4. This questionnaire is to ask what affects the susceptibility of your disease. Please indicate to what extent you agree or disagree.

Scale 1~5, 1. I completely agree, 2. 1 agree, 3. I am neutral, 4. I don’t agree, 5. I don’t agree at all.

1. Stress and things to worry about
2. Virus and Bacteria
3. Accident, injury, bone fracture, traumatic injury, operation
4. Food and eating habit
5. Inappropriate medication
6. Heredity
7. Environment pollution
8. Luck, by chance
9. My own behavior
10. Negative thinking
11. Family problem
12. Overwork
13. Own emotion
14. Age
15. Drinking habit
16. Smoking habit
17. Personality
18. Immune disorder
