# Supplementary material for: Effectiveness of personal genomic testing for disease-prevention behavior when combined with careful consultation with a physician: a preliminary study
Source: BMC Res Notes. 2018 Apr 3;11:223. doi: 10.1186/s13104-018-3330-9 (PMC5883259; doi:10.1186/s13104-018-3330-9)
Supplement: Supplementary file 3 — Additional file 3. Used PGT. [file 13104_2018_3330_MOESM3_ESM.docx]

**Additional File 3**

Used PGT;

<https://www.greenchord.jp/gene>

60 phenotypes including;

esophageal cancer, gastric cancer, colon cancer, pancreatic cancer, lung cancer, basal cell carcinoma, breast cancer, ovarian cancer, prostate cancer, testicular cancer, bladder cancer, glioma, thyroid cancer, chronic lymphocytic leukemia, type 1 diabetes, type 2 diabetes, obesity, essential hypertension, amyotrophic lateral sclerosis, Alzheimer’s disease, multiple sclerosis, glaucoma, age-related macular degeneration, myopia, ischemic heart disease, atrial fibrillation, brain aneurysm, chronic obstructive pulmonary disease, asthma, Crohn’s disease, ulcerative colitis, non-alcoholic steatohepatitis, primary biliary cirrhosis, hepatitis C, cholelithiasis, atopic dermatitis, male pattern alopecia, rheumatoid arthritis, systemic lupus erythematosus, gout, osteoarthritis, Behcet’s disease, osteoporosis, chronic kidney disease, urolithiasis, Graves’ disease, endometriosis, essential tremor, sarcoidosis, restless legs syndrome, psoriasis, alcohol flush, lactose intolerance, nicotine dependency, bitter taste perception, hemochromatosis, statin response, and warfarin response.
