# Supplementary material for: Effectiveness of personal genomic testing for disease-prevention behavior when combined with careful consultation with a physician: a preliminary study
Source: BMC Res Notes. 2018 Apr 3;11:223. doi: 10.1186/s13104-018-3330-9 (PMC5883259; doi:10.1186/s13104-018-3330-9)
Supplement: Supplementary file 4 — Additional file 4. Follow-up Questionnaire. [file 13104_2018_3330_MOESM4_ESM.docx]

**Additional File 4**

Follow-up Questionnaire;

１．Do you recall the results of the genetic test of yourself?

２．Have you been consulted regarding the results by any experts, e.g. physician?

３．Have you changed your life-style behavior?

If your answer in 3 is yes, please answer the following questions from 4 to 8.

４．Have you become more conscious about the quality and quantity of your daily food?

５．Have you become to do more daily exercise?

６．Have you become to reduce taking alcohol and/or smoking?

７．Have you started any healthy custom, such as taking supplementary diet?

８　Tell us what you started to do for your health if any rather than above.

９．Free comments
