# Supplementary material for: Effectiveness of personal genomic testing for disease-prevention behavior when combined with careful consultation with a physician: a preliminary study
Source: BMC Res Notes. 2018 Apr 3;11:223. doi: 10.1186/s13104-018-3330-9 (PMC5883259; doi:10.1186/s13104-018-3330-9)
Supplement: Supplementary file 5 — Additional file 5. Answer summary for Q3 section of the Pre- and post-PGT questionnaire. [file 13104_2018_3330_MOESM5_ESM.docx]

**Additional File 5**

Answer summary for Q3 section of the Pre- and Post-PGT questionnaire;

‘What would affect your future health or illness in ten years’ time?’

- asking the extent the subject agrees for each.
